# Common pathogenesis of early and late preeclampsia: evidence from recurrences and review of the literature

**DOI:** 10.1007/s00404-023-07217-z

**Published:** 2023-09-23

**Authors:** Svitlana Arbuzova

**Affiliations:** 1 Eastern-Ukrainian Center for Medical Genetics and Prenatal Diagnosis, Mariupol, Kiev, Ukraine; 2 Institute of Health Research, University of Exeter, Studio 3.4, Block M, Birks Hall, New North Road, Exeter, Devon, EX4 4GH England, UK

**Keywords:** Preeclampsia, Preterm, Term, Recurrence, Pathogenesis

## Abstract

**Objective:**

To investigate whether there is an association between the gestational age at the onset of preeclampsia in recurrent cases and the gestational age at the onset of preeclampsia in previous pregnancies.

**Methods:**

This retrospective nested case–control study was designed to investigate whether gestational age at diagnosis and at delivery in recurrent cases of preeclampsia correlates with gestational age at diagnosis and delivery in the previous cases of preeclampsia in the same individuals. The database of a Ukrainian research network was used to find patients with the diagnosis of preeclampsia between 2019 and 2021. The database was further queried to identify those with a history of preeclampsia in a previous pregnancy. The comparison was made using the Pearson correlation coefficient.

**Results:**

One hundred and three patients who were diagnosed with preeclampsia were identified. Of those, 15 had recurrent preeclampsia, 2 of whom had preeclampsia in 2 previous pregnancies. There was no statistically significant correlation: based on gestational age at delivery *R* = − 0.28 (*P* = 0.30; 95% confidence interval (− 0.69 to 0.28) and based on gestational age at the time of diagnosis *R* = − 0.14 (*P* = 0.62; − 0.60 to 0.41).

**Conclusion:**

Our data do not find an association between the gestational age of recurrent preeclampsia and preeclampsia diagnosed in a previous pregnancy. This supports the idea that there is single pathogenesis for preeclampsia regardless of the gestational age. It suggests that there are variations in the course of preeclampsia that may be determined by the capacity of the compensatory mechanisms.

## What does this study add to the clinical work

**Table:** 

Thе study demonstrates the lack of association between the onset of preeclampsia in recurrent cases, indicating a common pathogenesis of both events.	
It suggests that variations in the course of preeclampsia can be determined by the capacity of the compensatory mechanism.	

## Introduction

Preeclampsia (PE) is a leading cause of morbidity and mortality among mothers and infants worldwide. Despite considerable research and recent development of prenatal screening, the problem is far from resolved.

From a practical perspective, preeclampsia with either the onset of symptoms or the need for delivery, before 37 weeks of gestation (‘preterm’) is considered a separate entity from term preeclampsia. This is mainly because early-onset preeclampsia is generally more severe and leads to an early delivery, often of a growth restricted fetus. It not only increases the risk for the mother but also for the fetus and the neonate. Moreover, in first trimester screening for preeclampsia, the deviation from the normal of both biochemical and biophysical markers is greater in cases that are destined to develop early-onset preeclampsia. In addition, aspirin prophylaxis is more effective in preventing cases of early-onset preeclampsia than those that develop at term [1].

However, there is an ongoing controversy over whether preterm and term preeclampsia have the same pathogenesis. This stems from a hypothesis that there are two types of preeclampsia, “placental” and “maternal” [2]. Although it was subsequently demonstrated that all forms of preeclampsia involve the placenta and there is no single feature that is unique to early- or late-onset cases, the opinion has persisted that the hallmark of early preeclampsia is abnormal placentation and that late-onset preeclampsia is largely due to maternal factors.

If there are maternal factors that determine whether preeclampsia presents early or not, this should be apparent from studies of multiple cases delivered by the same woman. However, there are several studies in the literature indicating no consistency in gestational age between pregnancies with preeclampsia in the same woman. The present study is designed to investigate this issue further based on a series of Ukrainian women with recurrent preeclampsia.

## Methods

This retrospective nested case–control study uses a subgroup of patients from a previously published study [3]. That study investigated additional clinical risk factors available at the time of first trimester multi-marker screening for preeclampsia. It included 103 women with preeclampsia between 2019 and 2021 at various gestational ages [3]. In the present study, the database of a Ukrainian research network was used to find which of the 103 cases had preeclampsia in 1 or more previous pregnancies.

The gestational age at diagnosis of preeclampsia and gestational age at delivery in the most recent pregnancy was compared the same parameters in the previous affected pregnancies. Any association was quantified by the Pearson correlation coefficient, *r* value, and the average was used for those with more than one previously affected pregnancy. The null hypothesis of no association is tested using a two-tail *T*-test with *P* < 0.05.

## Results

Fifteen of the 103 cases investigated had recurrent preeclampsia. In 13 cases, only 1 of the previous pregnancies was affected. The remaining two had two previous pregnancies with preeclampsia. Of the 15 cases of recurrent preeclampsia, 3 (20%) were diagnosed at term (≥ 37 weeks’ gestation). A similar proportion (25%) of term preeclampsia was found in the 88 non-recurrent cases. Based on gestation at delivery, the proportions were also similar: 33% (5/15) and 41% (36/88), respectively.

**Fig. 1:**
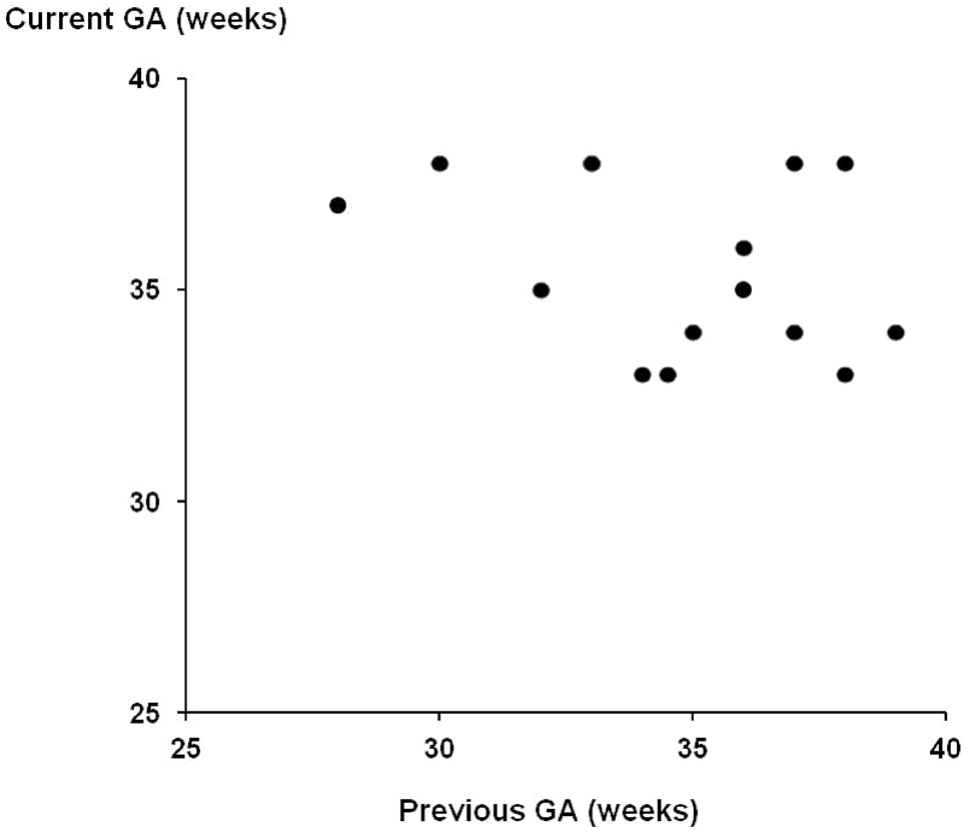
Scatter plot comparing gestational age at delivery in previous PE pregnancies and the current PE pregnancy

**Fig. 2:**
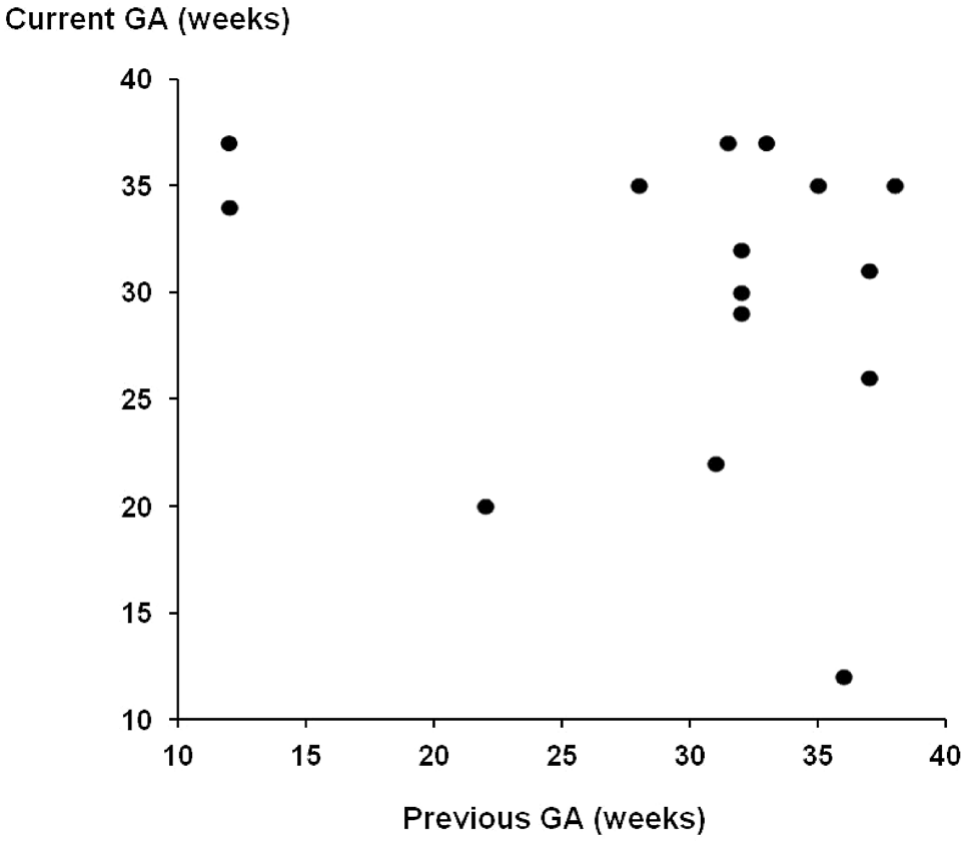
Scatter plot comparing gestational age at diagnosis in previous PE pregnancies and the current PE pregnancy

Figure 1 is a scatter plot of the gestational ages at the previous and current affected pregnancies. Based on the gestation at delivery, the *r* value was − 0.28 (*P* = 0.30) and 95% confidence interval (− 0.69 to 0.28). Figure 2 uses instead the gestation at diagnosis with preeclampsia. The *r* value was − 0.14 (*P* = 0.62) and 95% confidence interval (− 0.60 to 0.41).

## Discussion

This small series from Ukraine shows no significant association in the gestational age of preeclampsia between affected pregnancies in the same women. These data are consistent with the findings from other studies in the literature [4–14] and taken together supports the concept of a single pathogenesis for preeclampsia regardless gestation.

There are 11 published studies that look at specific ranges of gestational age in recurrent preeclampsia and the findings are summarized in Table 1. All of them reported the distribution of gestational age using broad groups, unlike our study which used exact gestations. In seven studies, the previous affected pregnancies were selected in a specific gestational range: < 24 weeks [6]; < 28 weeks [4]; < 34 weeks [5, 7, 12]; 34–37 weeks [8]; and > 37 weeks [9]. In the recurrent cases, the distribution of gestation, again in broad groups, was shown to be outside the range of the previous cases. In four studies, the distribution in broad gestational groups was given for the previous cases; three examined any association with the same groups in the recurrences [10, 11, 13], while one simply gave the distribution in the recurrences.

**Table 1:** Information on gestational age of diagnosis or delivery in 11 studies of preeclampsia recurrences

Study	Summary of design and findings
[4]	125 women with second trimester preeclampsia diagnosis followed up for 5.5 years on average. 110 recurrences: 35 (32%) in the second trimester, 35 (32%) at 28–36 weeks, and 40 (36%) at term
[5]	120 women with preeclampsia resulting in delivery < 34 weeks. 30 recurrences: 3 (10%) delivered < 34 weeks, 9 (30%) 34–37 weeks and 18 (60%) at term
[6]	20 women with preeclampsia < 24 weeks. 12 recurrences: 7 (58%) at 36–41 weeks, and 5 (42%) at 32–34 weeks
[7]	380 women with hypertensive disorder < 34 weeks including 183 preeclampsia, 10 eclampsia and 122 HELLP syndrome. Recurrences in 211 with subsequent pregnancies: 54 with preeclampsia—23 (64%) < 34 weeks, 14 (47%) 34–37 weeks, 17 (12%) at term and 18 with HELLP syndrome—9 (25%) with delivery < 34 weeks, 5 (17%) 34–37 weeks, 4 (3%) at term
[8]	425 women with hypertensive disorder at 34–37 weeks including 204 preeclampsia, 15 eclampsia and 104 HELLP syndrome. Recurrences in 189 with subsequent pregnancies: 96–8 (8%) with delivery < 34 weeks, 9 (9%) 34–37 weeks, 79 (82%) at term
[9]	503 women with hypertensive disorder > 37 weeks. 120 recurrences: 10 (8%) with delivery < 34 weeks, 5 (4%) 34–37 weeks, 105 (88%) at term
[10]	92 women with a history of preeclampsia of whom 55 had a recurrence within 4 years. Recurrence was similar in cases occurring < 34 weeks (65%) and ≥ 34 weeks (53%), although more likely to be < 34 weeks—94% of women with previous early onset and 57% with late onset
[11]	13 women with recurrent preeclampsia: 9 occurring < 34 weeks in previous pregnancy—6/10 (60%) had < 34 weeks in subsequent pregnancies. All 4 occurring ≥ 34 weeks had < 34 weeks in subsequent pregnancy
[12]	59 women with preeclampsia < 34 weeks. 13 recurrences: 6 (46%) < 34 weeks, 3 (23%) 34–37 weeks, and 4 (31%) at term
[13]	22,473 preeclampsia cases, no association between gestation of onset in previous and subsequent pregnancies. Proportion ≥ 34 weeks in subsequent pregnancy higher if < 34 weeks in previous pregnancy compared with ≥ 34 weeks. The risk of late preeclampsia in a second pregnancy was higher among women with history of early preeclampsia than among women with a history of late preeclampsia
[14]	5541 women with preeclampsia and 1662 recurrences. The proportion with delivery < 34 weeks is similar in the previous (7.8%) and the recurrent cases (8.8%)

Neither our series nor any studies in which the timing of onset was available [4–14] found an association between gestational age and disease recurrences, suggesting that the etiology of preterm and term preeclampsia is unlikely to be different.

### Limitations of the study

The present study is the first to report on the correlation between exact gestational age at diagnosis or delivery between previous and recurrent cases of preeclampsia. The finding of no statistically significant correlation may be due to the small sample size. However, this finding is consistent with the lack of association in grouped gestational data in other much larger studies. One further possible limitation is that the preeclampsia cases in the Ukraine study were predominantly preterm, whereas the population incidence of preterm preeclampsia in most countries is much lower than the incidence at term. This excess may have been the result of selection bias but should not have influenced the correlation with gestation in previous affected pregnancies.

### Arguments for and against a common pathogenesis for preterm and term preeclampsia—a review of the literature

The view that preterm and term preeclampsia have different pathogenesis has been widely accepted since the seminal paper of Roberts and Redman in 1993 [15]. This was subsequently reinforced by the proposition of Ness and Roberts that there are two types of preeclampsia, “placental” and “maternal”, the former linked to early onset (EOP, < 34 weeks) and the latter to late onset (LOP, ≥ 34 weeks) disease [2].

However, further studies have shown that changes in the placenta, though more common in EOP, also occur in LOP. In a large nested case–control study comprising 910 pregnancies with preeclampsia and more than 7000 controls, the frequency of pathologic findings consistent with placental hypoperfusion in the cases with preeclampsia decreased with gestational age at delivery from 75% at 25–26 weeks, to 55% at 33–34 weeks, and to 35% at term [16]. Furthermore, even at term, the frequency was approximately double that in controls.

Not all studies have found significant placental differences between late and early preeclampsia. The high rate of vascular lesions related to maternal underperfusion, in more than 50% of placentas, was shown to be typical for both preterm and term preeclampsia [17]. Some studies found that placental abruption, a well-described complication associated with preeclampsia, did not differ between EOP and LOP [18, 19]. No difference was found in the severity of decidual vascular disease and placental thrombi. The incidence of placental infarction was less common in EOP—48% (26/54) compared to LOP—61% (76/124) [20].

Moreover, it has not been possible to identify a single gestational age cutoff for the presence of placental lesions indicative of underperfusion which completely discriminates EOP from LOP. Thus, in 2019, the concept of “maternal” and “placental” preeclampsia was updated and it was proposed that all forms of preeclampsia involve the placenta [21].

A retrospective study of placental phenotypes in 230 women with EOL and 261 with LOP showed that the frequency of decidual arteriopathy, patterns of chronic hypoxic placental injury, villous infarction, excessive number of extravillous trophoblasts, and intervillous thrombi was considerably higher in both types of preeclampsia than in a control group. While hypoxic lesions and placental injury patterns were more common in EOP, associated with severe clinical outcomes, shallow placental implantation occurred at the same frequency in EOP and LOP, which is evidence of an underlying common pathological mechanism in both subgroups of preeclampsia [22]. In fact, there are no placental changes unique to preterm or term preeclampsia.

It has been proposed by Verlohren et al. [23] that term preeclampsia is pathologically heterogeneous. Of 21,677 women with complete date and outcome in their clinical study, who underwent uterine artery Doppler ultrasound examination, there were 1262 cases of preeclampsia. While early-onset preeclampsia was associated with more small for gestational age (SGA) and fewer large for gestational age (LGA) births, a higher prevalence of both LGA and SGA was shown in term preeclampsia suggesting its mixed etiology, partly analogous to early-onset preeclampsia with SGA and partly distinct. The authors assumed that a bimodal skewed distribution of birth weight in term preeclampsia could explain the weaker relationship between the mean resistance index and the maternal serum anti-angiogenic and pro-angiogenic factors (sFlt-1/PlGF) ratio compared with preterm preeclampsia.

The same conclusion that preeclampsia at term is heterogeneous and can be classified into two types was made by Chaiworapongsa et al. [24] who analyzed angiogenic and anti-angiogenic factors based on a longitudinal nested case–control study comprising 151 women and a case series of 452 women. An abnormal PlGF/sFlt-1 ratio was present in nearly all women with preterm preeclampsia -90% and 98%, and in 39% and 55% in those with term preeclampsia. Thus, the majority of women with early preeclampsia have an abnormal angiogenic profile, yet it is altered in half of women with late preeclampsia.

Although there is no single feature that is unique to preterm or term preeclampsia, the view remains that these two subtypes have distinct pathogenesis. Moreover, in a recent publication Roberts et al. now argue for the existence of several different subtypes: preterm/term, moderate/severe, and categorized according to the involvement of maternal organs [25].

Dividing preeclampsia into categories and the search for different causes depending on the gestational age at presentation may not be useful. Most diseases, despite fundamental underlying characteristics, present with a spectrum of symptoms in which compensatory mechanisms play a role. However, consideration of the processes of compensation and decompensation has never received its due attention in preeclampsia.

### Role of adaptation, compensation, and decompensation in preeclampsia

For any disorder or pathological condition, there are general processes that are modified through individual responses. Hence, the pathological condition is a stereotype but the disease itself is individual, characterized by common features which are transformed via compensatory mechanisms.

There are no changes in the placenta during preeclampsia which are not, to some degree, also present in normal pregnancy. For example, in a retrospective cohort study, including placental samples from 944 women who delivered at term without obstetrical complications, maternal and fetal vascular lesions of malperfusion were detected in 36% and 20%, respectively [26].

While preeclampsia is accompanied by considerable placental apoptosis, it is known that as the placenta grows and gestation advances, trophoblast apoptosis increases even in a normal pregnancy [27]. However, adaptive mechanisms make it possible to successfully cope with this in the majority of physiological pregnancies.

Compensation is very similar, but not identical, to adaptation. In the case of pregnancy, adaptation overlaps with compensation for balancing maternal–fetal functions. If compensation fails, then at one stage or another a disease occurs. The pathological state is always determined by the resilience of compensatory mechanisms, which are not limitless. There can often be a diminished level of compensation present, by which mild preeclampsia might be characterized, and a risk of decompensation, examples of which might be in HELLP syndrome and severe preeclampsia.

There is considerable evidence pointing to compensatory mechanisms that are more pronounced in term preeclampsia. Signs of branched angiogenesis have different intensities in the early and late forms but are more pronounced in late preeclampsia indicating a compensatory process [28]. This degree of compensation of the tissue in the area of the placental implantation site, which is exposed to chronic hypoxia, is typical for term disease but is not expressed in preterm preeclampsia [29]. Based on the pattern of placental findings in early and late preeclampsia, Moldenhauer et al. [19] also suggested that in cases that proceeded to term there could be compensatory mechanisms to enable adequate uteroplacental blood flow and prevent the disease becoming severe. Those patients who develop preterm preeclampsia may lack such compensatory mechanisms due to failure of vascular adaptation early in pregnancy.

Certain protective mechanisms in late preeclampsia have also been demonstrated using untargeted proteomics: apolipoprotein E (APOE), apolipoprotein C4 and C2 (APOC4-APOC2) were all significantly increased in LOP, whereas they were marginally increased in EOP. Although there was some overlap APOE, widely known for its protective role in atherosclerosis, it is more relevant to LOP [30]. It is well accepted that oxidative stress and mitochondrial dysfunction are key factors in the pathophysiology of preeclampsia [31]. Mitochondrial changes are characteristic of both early and late preeclampsia [32] and placental total oxidant levels are markedly elevated and total antioxidant levels are decreased in both EOP and LOP placentas [33].

At the same time, it has been clearly shown that placentas from preeclamptic pregnancies that reached term showed multiple adaptations in mitochondrial function and related processes that were only minimally observed in preterm preeclampsia [34]. In placentas from term preeclamptic pregnancies, there was an increase in total antioxidant activity. In contrast, placentas with preterm preeclampsia have been found to have reduced antioxidant function. Mitochondrial complexes II and III were increased in placentas with term preeclampsia but did not change in placentas with preterm preeclampsia compared to controls. In addition, there was an overall increase in placental mitochondrial respiration in term placental preeclampsia*.* Thus, although placentas from term and preterm preeclampsia were found to have elevated levels of reactive oxygen species such as H_2_O_2_ in term preeclampsia, a compensatory mechanism is activated with an increased total antioxidant activity. However, as shown in this study, placental tissue in the presence of preeclampsia at term had higher levels of mitochondrial respiration but there was a decrease in reserve capacity, indicating that the mitochondria were functioning closer to their maximum. The latter is a good example of the cost of compensation; exceeding the limit of its capacity may lead to decompensation.

The concept that the capacity of the compensatory mechanism varies and can determine differences in the course of preeclampsia, refutes the argument of Roberts et al., which states that there are several preeclampsia subtypes [25]. In particular, the authors seek to substantiate their view by referencing the fact that among women diagnosed with preeclampsia there are those with an indolent disorder and slow changes, and those who exhibit minimal signs and symptoms and who suddenly become critically ill. But compensation, though it may proceed successfully for some time, may be fragile and is liable to a sudden collapse. This decompensation is often characterized by just such an acute change in state as has been repeatedly proven in various diseases.

Another argument by Roberts et al. justifying the presence of many subtypes of preeclampsia is that the principal organs involved in preeclampsia vary between cases [25]. However, with the uniformity of the symptoms that determine the disease, heterogeneity of some signs and symptoms is a well-known medical phenomenon. Besides, when compensation mechanisms in the organs that dominate in this process are overstretched, a result may be a cascade of events affecting the function of organs that are not directly involved.

### Further research testing the hypothesis of compensation in preeclampsia

One possible approach is to develop measurable indicators of resilience and compensatory mechanisms, which might potentially lead to treatment.

One such indicator may be bile acid levels. Impaired hepatobiliary function in pregnancies with preeclampsia is well documented and in the recent Ukraine study, a very high incidence of cholelithiasis was found in women with preeclampsia and their mothers [3]. The toxicity of the increased level of bile acid can directly damage placental tissue causing changes reminiscent of those in preeclampsia, inducing oxidative stress and trophoblast cells apoptosis, and affect fetal and placental blood circulation [35].

Another indicator could be bilirubin. It is an end product of heme catabolism and represents the principal bile pigment. It is also an endogenous antioxidant. As such, it may have a protective effect in oxidative stress-mediated diseases, which has been shown in several animal and clinical studies [36]. Bile acids appear to decrease intracellular bilirubin levels [37]. This is pertinent as low levels of bilirubin have been shown to be associated with a worsened prognosis for preeclampsia [38]. Therefore, an increase in the bile acid/bilirubin ratio may be an informative predictor of the disease course and a marker of increased risk of preeclampsia.

## Conclusion

Neither our data, nor any studies where exact gestational ages were available, found an association between gestational age at preeclampsia recurrence and gestational age at diagnosis in the previous pregnancy. This supports the idea that there is a single pathogenesis for preeclampsia. It also suggests that variations in the course of preeclampsia may be determined by the strength of the compensatory mechanisms.

## Data Availability

The research data supporting this publication are available on request from the University of Exeter's institutional repository at: 10.24378/exe.4727.
